# Restoring the supply lines: removing roadblocks to fatty acid uptake enhances T cell-driven cancer fight

**DOI:** 10.1038/s41392-025-02151-9

**Published:** 2025-02-14

**Authors:** Jeremy G. Baldwin, Caio R. F. Silveira, Luca Gattinoni

**Affiliations:** 1 Division of Functional Immune Cell Modulation, Leibniz Institute for Immunotherapy, Regensburg, Germany; 2 University of Regensburg, Regensburg, Germany; 3 Center for Immunomedicine in Transplantation and Oncology, University Hospital Regensburg, Regensburg, Germany

**Keywords:** Immunotherapy, Tumour immunology, Metabolic engineering

In a recent study published in *Nature*, Hwang et al.^1^ show that the tumor microenvironment (TME) constrains tumor-infiltrating CD8^+^ T cell responses by curtailing fatty acid uptake and utilization through endoplasmic reticulum (ER) stress-induced transgelin 2 (TAGLN2) suppression. Overexpression of TAGLN2 in CD8^+^ T cells bypasses the ER stress response, restoring fatty acid shuttling, bioenergetic fitness, and antitumor efficacy.

General Dwight D. Eisenhower once stated that battles, campaigns, and even wars are won or lost primarily because of logistics. Getting the right resources where they need to be at the right time is crucial in determining success on the battlefield. The fight against cancer demands no less similar requirements. CD8^+^ T cells are the front-line soldiers tasked to target and eliminate cancer cells. However, when operating behind enemy lines in the TME –a hostile territory where oxygen and nutrients are scarce– T cells are forced to scavenge and even compete directly with cancer cells for resources, including fatty acids, to sustain their survival and function.

**Fig. 1:**
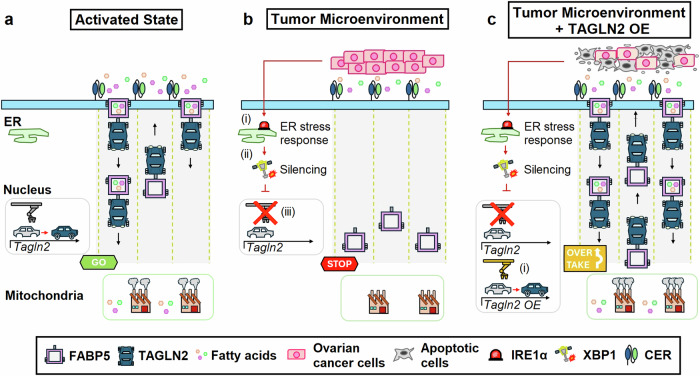
Transgelin 2 overexpression in chimeric endocrine receptor-engineered T cells restores fatty acid uptake and enhances antitumor function. **a** Once activated, chimeric endocrine receptor (CER)-engineered T cells upregulate transgelin 2 (TAGLN2) expression. TGLN2 facilitates the trafficking of fatty acid-binding protein 5 (FABP5) to the membrane surface of T cells, enabling the import and intracellular trafficking of exogenous fatty acids to mitochondria where they are used for energy generation through β-oxidation. **b** In the ovarian cancer tumor microenvironment, endoplasmic reticulum (ER) stress induces (i) activation of inositol-requiring enzyme 1α (IRE1α), which (ii) mediates the splicing of the transcription factor X-box-binding protein 1 (XBP1) into its active isoform, which then (iii) represses *Tagln2* expression. Silencing of TAGLN2 prevents FABP5 surface localization, inhibiting fatty acid uptake and reducing metabolic fitness of CER-engineered T cells. **c** Overexpression (OE) of TAGLN2 (i) bypasses ER stress-mediated silencing, enhancing fatty acid uptake, metabolic fitness, and antitumor function of CER-engineered T cells

In this study, Hwang et al.^1^ demonstrate that TAGLN2 interacts with fatty acid-binding protein 5 (FABP5) to facilitate its localization on the cell surface. Together, these two proteins cooperate to optimize fatty acid import into CD8^+^ T cells for lipid metabolism and β-oxidation within mitochondria (Fig. 1a). However, the tumor employs a clever strategy to disable this vital supply line. The group discovered that the TME silences TAGLN2 expression, effectively shutting down fatty acid uptake in CD8^+^ T cells. Several putative transcription factors binding sites associated with endoplasmic reticulum (ER) stress were identified at the promoter *Tagln2* locus. Through the use of transgenic deficient mice, the authors identified a cascade of molecular events mediating *Tagln2* repression: (i) activation of inositol-requiring enzyme 1α (IRE1α) which (ii) mediates the splicing of the transcription factor X-box-binding protein 1 (XBP1) into its active isoform, and (iii) enables XBP1 to bind tightly to the promoter regions of *Tagln2* (Fig. 1b). Remarkably, the researchers were able to rescue ER stressed CD8^+^ T cells by overexpressing TAGLN2, which re-enabled lipid uptake, resulting in increased mitochondrial respiration, cytokine production and cytotoxicity.

To further explore the therapeutic potential of TAGLN2 overexpression, Hwang et al. subcloned the *Tagln2* gene downstream of a chimeric endocrine receptor (CER) construct, which targets follicle-stimulating hormone-receptor positive (FSHR^+^) ovarian tumors. The resulting CER-TAGLN2 T cells infiltrated more efficiently the metastatic lesions and effectively controlled disease progression in a mouse model of high-grade serous tubo-ovarian cancer (HGSC) that harbors the most frequent genetic mutations detected in patients with high-grade serous ovarian cancer. Tumor-infiltrating CER-TAGLN2 T cells maintained high TAGLN2 expression and surface levels of FABP5 compared to control CER T cells present at the same tumor sites. Interestingly, PD-1 blockade did not boost the therapeutic effects of CER-TAGLN2 T cells, leading the authors to conclude that TAGLN2 silencing impaired the ability of CER T cells to control advanced ovarian tumors. It is important to emphasize that, while the authors demonstrate an improvement in fatty acid uptake –potentially contributing to enhanced CD8^+^ T cell metabolism in the TME– it remains unclear whether this mechanism directly underpins the improved therapeutic outcomes observed in CER-TAGLN2 T cells. Beyond fatty acid metabolism, TAGLN2 is known to facilitate T cell adhesion to target cells and immune synapse stabilization,^2^ which could also contribute to the observed effects. To better understand the specific contributions of fatty acid metabolism, overexpression of TAGLN2 in FABP5-deficient CER T cells could serve as an important control, helping to clarify whether TAGLN2’s antitumor effect is primarily driven by lipid metabolism or by other mechanisms.

Given the limited clinical success of T cell therapies in solid tumors, particularly in metastatic ovarian cancer, there is an urgent need to develop strategies to fortify T cells and enhance their ability to thrive in the hostile TME. Other groups have explored complementary approaches to mitigate ER stress in T cells. For instance, pharmacological inhibition of XBP1 or targeting other downstream sensors of ER stress, such as C/EBP homologous protein (CHOP) and protein kinase R-like endoplasmic reticulum kinase (PERK)/endoplasmic reticulum oxidoreductase 1 alpha (ERO1α) have been shown to improve the effector function of tumor-reactive CD8^+^ T cells.^3,4^ The insightful work of Hwang et al. adds another exciting piece to the puzzle of overcoming the challenges of ER stress in TME. It will be interesting to see whether these different interventional strategies could be combined and act synergistically to further extend T cell efficacy against ovarian cancer as well as other solid tumors. Future work should also focus on elucidating the nature of the signals in the TME that trigger the ER stress response that leads to the downregulation of TAGLN2, as well as determining if the same mechanisms of TME-mediated immunosuppression affect other immune cell subsets.

Lastly, when considering military strategies, it is essential to take a holistic view of the whole vertical supply chain rather than focusing solely on transportation elements. If, for example, the TME also damages or destroys the cells’ factories (i.e. mitochondria), the cells may be unable to effectively utilize resources even if the supplies reach their destination. In such cases, combining TAGLN2 overexpression with emerging technologies, such as mitochondrial transfer or targeted therapies that protect/promote mitochondria biogenesis, could restore or enhance fatty acid oxidation, enabling T cells to achieve improved antitumor efficacy.^5^

In summary, Hwang et al. have demonstrated the battle against cancer is as much a logistical challenge as it is a fight of firepower, highlighting the importance of securing metabolic resources for T cells to ensure success when combating cancer. By ensuring that the supply lines of fatty acids remain open, we can empower T cells to perform their vital role on the hostile front lines of the tumor microenvironment, ultimately paving the way for more effective cancer immunotherapies for patients.
